# Successful desensitization in a patient with Von Willebrand´s disease and anaphylaxis to factor VIII/VW

**DOI:** 10.1186/2045-7022-4-S3-P61

**Published:** 2014-07-18

**Authors:** Maristela Olival, Mara Felix, Monica Soares, Marilia Renni, Silmara Montalvao, Luis Ensina, Mariana Castells

**Affiliations:** 1 Hematological State Institute “Arthur de Siqueira Cavalcanti”– HEMORIO, Rio de Janeiro, RJ, Brazil, Department of Hematology, Clinical Hematology Divi, Brazil; 2 Federal Hospital of Servidores do Estado, Department of Pediatrics, Allergy and Immunology, Brazil; 3 University of Campinas, Hematology and Hemotherapy Center, Brazil; 4 Federal University of São Paulo, Division of Clinical Immunology and Allergy, Brazil; 5 Brigham and Women’s Hospital, Department of Medicine, Division of Rheumatology, USA

## Background

Von Willebrand´s disease (VWD) is the most common congenital disorder of hemostasis, characterized by deficient or defective von Willebrand factor. Patients are treated by intravenous replacement of factor VIII/VW (FVIII/VW) when needed, for prophylaxis before surgical procedures. Anaphylactic reactions to FVIII/VW are rare and desmopressin (DDAVP) can be used as an alternative.

## Materials and methods

We report a case of a 16-year-old female, with VWD and no personal or family history of atopic disease, that was referred to our unit after 4 episodes of facial, upper and lower extremities urticaria, 30 minutes after infusion of FVIII/VW. She has been treated with the factor since 2000, and had her first allergic reaction in 2004. After the first episode, she received pretreatment with oral dexchlorpheniramine and hydrocortisone IV. The last episode was in March 2013, when she had laryngeal edema, which required epinephrine. The factor was replaced by DDAVP, but she developed itching, flushing and hypertension. We evaluated the patient using the ENDA questionnaire and skin tests. Skin prick test (SPT) to FVIII/VW (Octavi® SDOptimum; Octapharma, Vienna, Austria) was performed at full-strength concentration (50 U/ml) with histamine as positive control and normal saline as negative control. Intradermal skin tests (ID) were performed with 1:1000, 1:100, 1:10 and 1:1 concentrations. The reactions were assessed at 15 minutes. Her mother served as control subject and gave informed consent before testing and desensitization. A desensitization protocol was designed for the patient. Briefly, intravenous infusions of FVIII/FVW were started at 0.01 U/kg and doses were doubled at 10-minute intervals initially and then at 15-minute intervals to reach a cumulative dose of 30 U/ml. She was pre-medicated with diphenhydramine 25 mg, ranitidine 50 mg and montelukast 10 mg.

## Results

SPT were negative in both patient and control subject. ID reactions at all dilutions were negative in control subject but it was positive at 1:1000 dilution in the patient (wheal of 8 mm). She developed an urticarial rash 4 hours after skin tests, treated with fexofenadine. Four days later, she underwent successful desensitization which was completed in 2 hours and 35 minutes.

## Conclusions

Rapid desensitization for FVIII/VW induced anaphylaxis should be considered in patients with VWD to allow for safe surgical procedures.

